# The evolutionary history of *Antirrhinum* in the Pyrenees inferred from phylogeographic analyses

**DOI:** 10.1186/1471-2148-14-146

**Published:** 2014-06-26

**Authors:** Isabel M Liberal, Monique Burrus, Claire Suchet, Christophe Thébaud, Pablo Vargas

**Affiliations:** 1 Real Jardín Botánico de Madrid (CSIC), Plaza de Murillo 2, Madrid E-28014, Spain; 2 Laboratoire Evolution et Diversité Biologique (EDB), UMR5174, CNRS - Université de Toulouse (UPS)- ENFA, 118 route de Narbonne, Toulouse, Cedex 9 31062, France

**Keywords:** *Antirrhinum*, Phylogeny, Phylogeography, Pyrenees, Quaternary

## Abstract

**Background:**

The origin and colonisation history after the Quaternary ice ages remain largely unresolved for many plant lineages, mainly owing to a lack of fine-scale studies. Here, we present a molecular phylogeny and a phylogeographic analysis of *Antirrhinum*, an important model system in plant biology, in the Pyrenees range. Our goal was to reconstruct the evolutionary and colonisation history of four taxa endemic to this region (*A. majus* subsp. *majus*, *A. majus*. subsp. *striatum*, *A. molle*, and *A. sempervirens*) by using a dense sampling strategy, with a total of 452 individuals from 99 populations whose collective distribution spans nearly the entirety of the Pyrenees and adjacent mountains.

**Results:**

Phylogenetic and phylogeographic analyses of the sequences of two plastid (*trn*S-*trn*G and *trn*K-*mat*K) regions revealed the following: (i) historical relationship between the Pyrenees and Iberia (but not with the Alps); (ii) the long persistence of populations in the Pyrenees, at least since the Late Pleistocene; (iii) three different colonisation histories for populations from the Western, Central, and Eastern Pyrenees; (iv) the deep phylogeographic separation of the eastern and western populations; and (v) the colonisation of southern France from the Eastern Pyrenees.

**Conclusions:**

The present study underlines the enormous influence of the glacial history of the mountain ranges on the current configuration of intra- and inter-specific genetic diversity in *Antirrhinum*, as well as the importance of periglacial areas for the survival of species during glacial periods of the Quaternary.

## Background

The three mountain systems (the Pyrenees, Alps, and Balkan mountains) located to the north of the major southern European peninsulas (Iberia, Italy, and Balkans) have played an important role in determining the current distributions of European biota, and they harbour exceptionnally high levels of biodiversity [1-3]. Their high elevation greatly influenced the climate-induced range shifts of many species during Quaternary climatic cycles [4-7], and local speciation promoted by isolation of populations in long-standing refugia and potential contact zones between postglacial recolonizing genetic lineages have been invoked to account for such diversity [8,9].

The Pyrenees are especially renowned as an important plant diversity hotspot because of their high floristic richness (around 3500 species and subspecies of vascular plants) [10,11], including considerable endemicity (about 4% of the plant species are endemic) and numerous plant associations [12,13]. Yet we still know relatively little about the processes that led to species accumulation within this region. Their east–west arrangement clearly acted as a latitudinal barrier for postglacial dispersal predominantly out of the Iberian Peninsula. Moreover, they served as an arena in which postglacial lineage contacts occurred [9]. While the valleys in other European mountain ranges were only partially covered by ice even during the coldest periods of the ice age glaciations (*e.g.* Balkan Mountains and Carpathians), the Alps and the Pyrenees were almost entirely covered by large ice sheets [14-17]. Such similarities in their glacial history may have resulted in analogous patterns of genetic structure of the plant species that inhabit the two east–west mountain ranges. Unfortunately, in contrast to the high number of studies that have focused on the evolutionary history of plant lineages in the Alps [see [18], few investigations have addressed the origin and colonisation history of Pyrenean plant species in a phylogenetic context at the appropriate spatial scale. Most of the previous phylogeographic studies that included the Pyrenean range have relied upon limited sampling strategies (but see [19]). Thus, the major patterns related to the origins and colonisation of Pyrenean plant lineages after the Quaternary ice ages remain largely unresolved.

The present study used *Antirrhinum* species from the Pyrenees to reconstruct the colonisation history of mountain plants in this region. Monophyly of this genus, an important model system in plant biology primarily distributed in the western Mediterranean basin, has been previously proved [20]. The Iberian Peninsula harbours the highest species diversity; however, a few *Antirrhinum* species are also found in other European areas, such as the Alps (*A. latifolium*) and Italy (*A. siculum*), or are distributed widely across the Mediterranean (*A. tortuosum*) despite the lack of any obvious long-distance dispersal syndrome. Historically, the number of *Antirrhinum* species has been the subject of taxonomic debate, resulting in different taxa circumscriptions. The difficulties in species delimitation, as well as the discrepancies between phylogenetic relationships and taxonomic classifications, have been interpreted as strong evidence for recent geographic speciation and extensive hybridisation since the Pliocene [21-24].

Four *Antirrhinum* taxa from two sections are endemic to the Pyrenees and pre-Pyrenees range. The pre-Pyrenees range is formed by a complex system of foothill ranges which stretches from the western side (influenced by the Atlantic regime) to the Mediterranean coast on the eastern end of the Pyrenees (commonly called sub-Pyrenees). This range mostly runs parallel to the Pyrenees along the Iberian side, but it also includes the Corbières Range, towards the eastern end on the French side, where the Pyrenees’s slopes descend rather abruptly. The four endemic taxa occur in different habitats throughout these mountain ranges, ranging from lowland areas (800 m) to high mountain environments (>2500 m). *A. molle* and *A. sempervirens* (sect. *Kickxiella*) inhabit limestone cliffs from medium to high altitudes, whereas *A. majus* subsp. *majus* and *A. majus* ssp. *striatum* (sect. *Antirrhinum*) occur mostly in lowland areas of the Eurosiberian woodlands (see Additional file 1 for further clarification of the different taxonomic treatments). Previous phylogeographic studies found that four of the eight main *Antirrhinum* lineages are distributed in northeast Iberia [22]. Thus, the Pyrenees along with the pre-Pyrenees mountains are one of the areas with the highest numbers of genotypes and lineages. Recent studies have also been conducted to understand the cause and the maintenance of the parapatric distribution of two of these taxa (*A. majus* subsp. *majus* and *A. majus* subsp. *striatum*), as well as the ecological process that occurs in narrow contact zones [25-27]*.* Thus, it seems reasonable to assume that the present-day distribution of *Antirrhinum* in the Pyrenees is the result of historical processes that have taken place across the mountain range and adjacent mountains during Quaternary times. We evaluate this possibility with molecular phylogenetic and phylogeographic analyses.

Based on previous phylogenetic and phylogeographic studies on plants (see Additional file 2), four hypotheses (not mutually exclusive) can be proposed for explaining the origin of *Antirrhinum* lineages that are found in the Pyrenees: (i) a predominantly southern (Iberian) origin; (ii) a predominantly northern (central European) origin; (iii) a long-standing isolation of Pyrenean populations; and (iv) recurrent secondary contacts between Iberian and central European lineages.

In this study, we first attempt to infer the historical connections between the Pyrenees and other geographic areas. We also investigate the genetic structure within the species and assess the patterns of genetic diversity within the Pyrenees region. By combining the results of these analyses, we explore potential processes that could explain the evolutionary history of *Antirrhinum* lineages throughout the Pyrenees.

## Results

### Phylogenetic analysis

Detailed features of the two sequenced cpDNA regions are summarized in Table 1 (GenBank accession numbers are shown in Additional file 3). The total aligned length of the combined *trn*S-*trn*G/*trn*K-*mat*K dataset of the 104 samples added to the 83 sequence matrix of Vargas et al. [22] was 1795 bp (excluding the outgroup). This revealed 82 variable sites of which 57 were parsimony-informative.

**Table 1:** Features of the two sequenced cpDNA regions

	***trn*S-trnG**	***trn*K-*mat*K**
Aligned length (bp)	537	1266
Ungapped length range	1-518	519-1764
Pairwise % identity	99.5	99.7
Variable characters	26	56
Parsimony-informative characters	21	36
Mean % G + C content	31.1	34.2

Characteristics of the *trn*S-*trn*G and *trn*K-*mat*K sequences obtained for *Antirrhinum* matrix (excluding outgroup).

**Figure 1:**
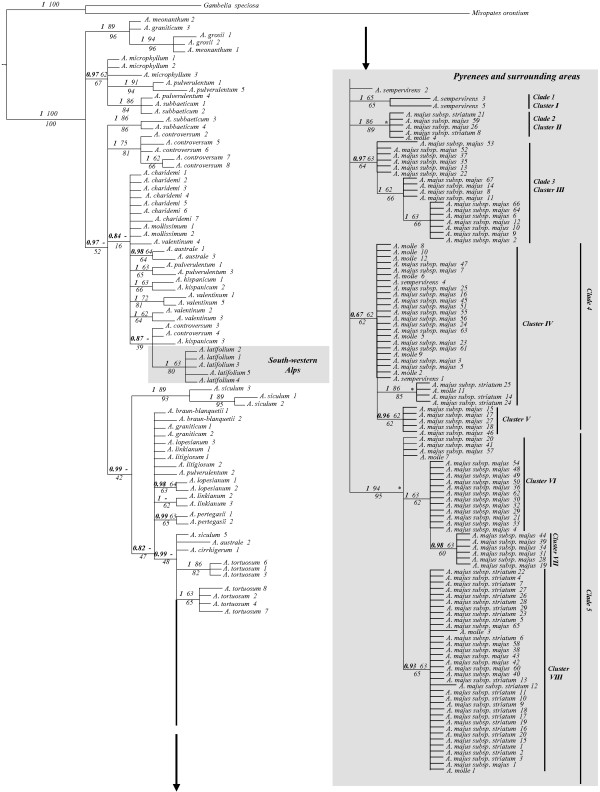
**Phylogenetic analyses of *Antirrhinum *based on 190 *trn*S-*trn*G/ *trn*K-*mat*K sequences.** The Bayesian tree is shown, in addition to values above branches for Bayesian posterior probabilities (PP) and parsimony bootstrap percentages (BP), values below branches indicate maximum likelihood bootstrap values (BS). A dash (-) above branches indicates disagreement between the maximum parsimony (MP) strict consensus tree and the Bayesian inference (BI) and maximum likelihood (ML) trees. Nodes with high support are indicated with an asterisk (*).

The 50% majority-rule consensus tree of the Bayesian analysis is shown in Figure 1. No conflicts are found between the Bayesian phylogenetic analyses for the *trn*K-*mat*K and *trn*S-*trn*G separate matrices (Additional files 4 and 5). The strict consensus tree of the MP (maximum parsimony) analysis was basically congruent with the ML (maximum likelihood) analysis, although with a lower resolution and support values (Figure 1). One subclade recognized under Bayesian posterior probabilities (*PP*) contained all *A. majus* subsp. *majus*, *A. majus* subsp*. striatum*, *A. sempervirens* and *A. molle* accessions (*PP =0.99*) together with the accessions of species from southern Iberia (*A. australe* and *A. cirrhigerum*) and those species widespread across the Mediterranean (*A. tortuosum* and *A. siculum*). No analyses supported the monophyly of Pyrenean samples. The five samples of *A. latifolium* from the South-western Alps were clustered together (*PP =1; ML-BS –* maximum likelihood bootstrap *– = 80*%*; MP-BP –* maximum parsimony bootstrap percentages *– = 63*%) and clearly unrelated to Pyrenean samples. All three phylogenetic analyses showed high support values for clade 5 (*PP =1.0; ML-BS = 95*%*; MP-BP = 94*%), which comprised samples primarily distributed in the Eastern Pyrenees.

### Estimation of divergence times

**Figure 2:**
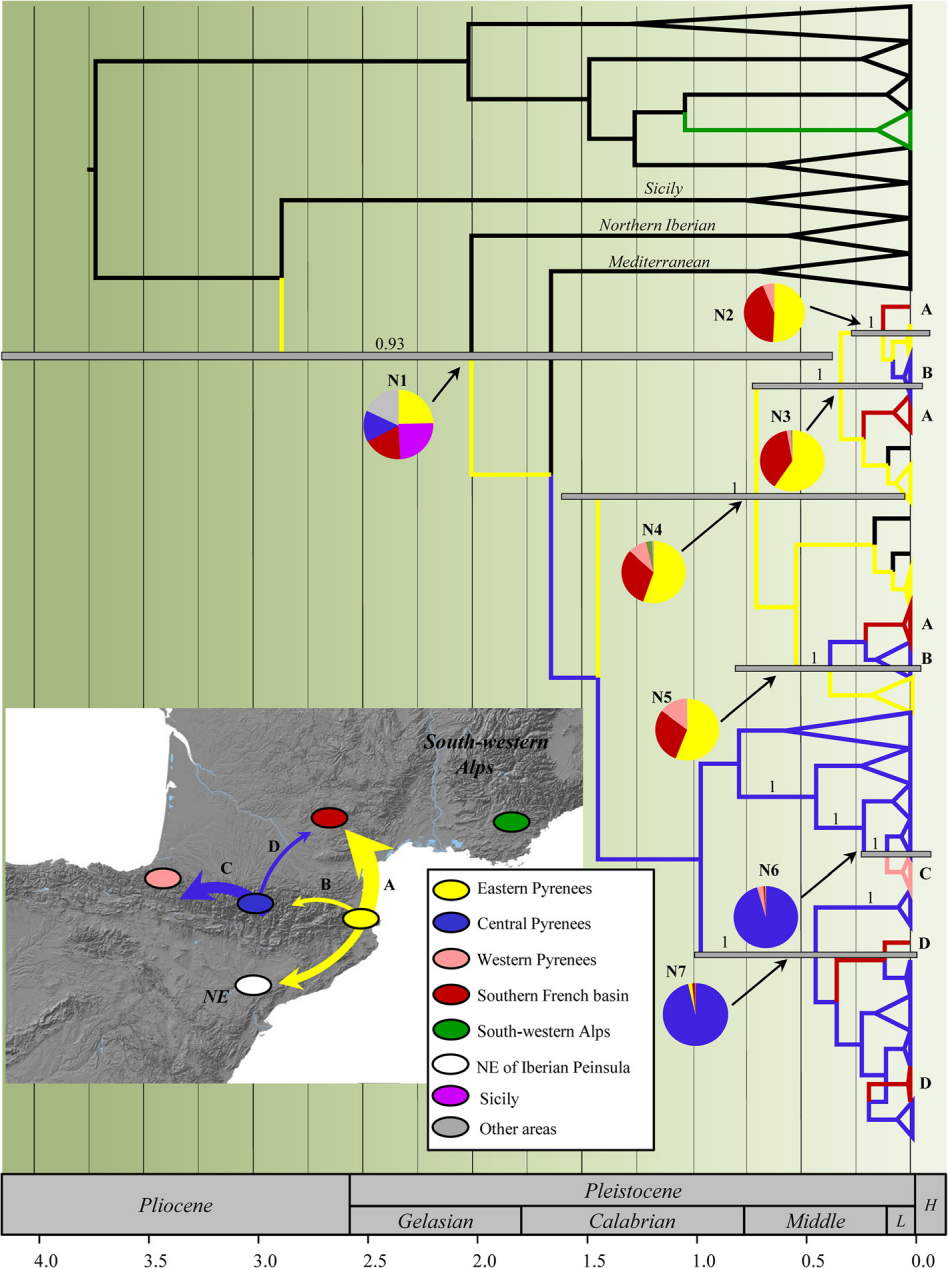
**Biogeographic reconstruction based on 190 concatenated *trn*S-*trn*G/ *trn*K-*mat*K sequences of *Antirrhinum*.** The tree summarizes the geospatial Bayesian analysis. Pie charts represent posterior probability distributions of ancestral states at well-supported nodes. Colonisation routes supported by a BF > 3 are shown on the map (see Additional file 6). The inset shows the study mountain range (Pyrenees and neighboring areas). Colors in ellipses and branchs represent the areas of origin. Arrows specify directionality in the colonisation route, inferred from well-supported nodes of interest in the geospatial Bayesian analysis. Arrow width indicates relative support of migrations. Node bars represent the 95% highest posterior density intervals for the divergence time estimates. Numbers above branches are Bayesian posterior probabilities. Abbreviations used: L, Late Pleistocene; H, Holocene.

The values of standard deviation of the uncorrelated lognormal relaxed clock (0.31) and coefficient of variation (0.33) for rate heterogeneity within our *trn*S-*trn*G/*trn*K-*mat*K dataset indicated low but significant rate heterogeneity among lineages (>0.1). Therefore the use of the uncorrelated clock was considered appropriate. Examination of the MCMC samples with Tracer 1.5 [28] confirmed adequate sample size, with ESS (effective sample size) values in the hundreds or thousands for both dating analyses. These also indicated adequate convergence and stationarity of the posterior probability distributions after discarding the burn-in. The topology of the maximum clade credibility (MCC) tree (Figure 2) was congruent with those of ML and MP analyses. The analysis revealed geographically structured lineages diverging since the Pliocene. A highly supported lineage (N1) containing samples of Northern Iberia (*A. braun-blanquetii*, *A. graniticum*, *A. lopesianum*, *A. linkianum*, *A. litigiosum*, *A. pulverulentum* and *A. pertegasii*), Mediterranean basin (*A. siculum*, *A. australe*, *A. cirrhigerum*, *A. tortuosum*) and the four Pyrenean species was estimated to split between 0.46 and 4.2 Ma – 95% Highest Posterior Density (HPD) – in the late Pliocene/early Pleistocene. A well-supported lineage primarily from the Eastern Pyrenees (N4) diverging between 0.13 and 1.7 Ma (100% HPD) was also observed. Additionally, we found highly supported sublineages of Pyrenean populations diverging in the Quaternary (see Figure 2).

### Ancestral area reconstructions

DPA analysis showed uncertainty in the geographic origin of the Pyrenean lineages; since several areas outside the Pyrenees showed similar probabilities (see Figure 2). Nevertheless, stronger support was found for the geographic origin of more recent subclades, leading to a robust reconstruction of dispersal events within and around the Pyrenees during the Pleistocene. Bayes factor (BF) tests (Additional file 6) for significant non-zero rates revealed a historically close connection between the Pyrenees and the Iberian Peninsula (BF = 21.93). In contrast, South-western Alps populations (where *A. latifolium* occurs) were not linked to the Pyrenees. The Central Pyrenees were the most likely ancestral location of western haplotypes (BF = 36.74). A colonisation episode involving a few populations may have also occurred from Central Pyrenees to the Southern French basin, as indicated by a well-supported connection between these two areas (BF = 10.45). However, the colonisation of the Southern French basin seems to have occurred mainly from the Eastern Pyrenees as indicated by a considerably higher Bayes factor value (BF = 404.17). Interestingly, a less supported link was also observed between Eastern and Central Pyrenees (BF = 4.13). These results suggest: (i) no close-relationship between Pyrenean lineages and those distributed in the South-western Alps; (ii) an origin of Pyrenean lineages during the Pleistocene; and (iii) recent dispersal episodes within the Pyrenees from two main spreading centers (Eastern and Central Pyrenees).

When the number of areas was reduced to four –Pyrenees and surrounding areas, South-western Alps, Iberian Peninsula (excluding the Pyrenees) and rest of Mediterranean areas– the inferred historical biogeographic scenarios from the S-DIVA (Statistical Dispersal-Vicariance Analysis) and DEC (Dispersal-Extinction-Cladogenesis) analyses were mostly congruent, but not very informative about the origin of the Pyrenean lineages (Additional files 7 and 8 respectively). However, the results agreed with a sister relationship between the South-western Alps lineage and an Iberian clade, indicating: (i) a historical connection between two distant areas and (ii) no recent relationship between the Alps and Pyrenean lineages.

### Haplotype data analysis

#### Antirrhinum network

The statistical parsimony analysis of the *Antirrhinum matrix* found 11 haplotype lineages, of which seven included samples from NE Iberia. In addition, five haplotype lineages from the Pyrenees were formed by 18 haplotypes (haplotypes 2, 9-12 and 52 - 63, see Additional file 9) obtained from the 99 populations collected across the Pyrenees and adjacent areas. Some missing haplotypes (extinct or not found) and one loop were retrieved for the whole genus. Most of the haplotypes were grouped primarily together within lineages I and II of northern Iberia (Additional file 9). The five samples collected in the South-western Alps provided two new haplotypes (haplotypes 64 and 65) with a tip position, which formed part of lineage VI from southeastern Iberia.

#### Pyrenees network

**Figure 3:**
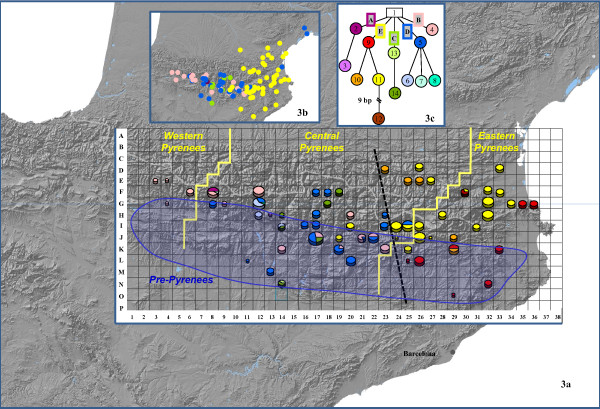
**Distribution of haplotypes and lineages based on 452 *trn*S-*trn*G sequences. (3a)** Haplotypes distributed across the Pyrenees and delimited by a grid of 10x10 km quadrats in which haplotypes of all the populations at the same quadrat are grouped together (pie charts). **(3b)** Distribution of cpDNA lineages. **(3c)** Haplotype network of Pyrenean populations. Labels on the lines connecting haplotypes represent the plastid lineages. The dashed black line represents the approximate location of the genetic boundary indicated by SAMOVA. Yellow lines delimit the historical phytogeographic areas (Western, Central and Eastern Pyrenees). The blue shadow represents the approximate location of the pre-Pyrenees range.

The haplotype network analysis of the *Pyrenees matrix* distinguished 13 haplotypes (hereafter named H1–H13) distributed into five lineages (hereafter named lineages A–E). The H12 resulted from a 9 bp deletion. Neither loops nor missing haplotypes (extinct or not found) were observed (Figure 3c). No species had exclusive haplotypes, except for *A. sempervirens* that had four (H2, H3, H7 and H8) (Additional file 10).

### Genetic structure and diversity

The geographical distribution of haplotypes and lineages is shown in Figure 3 (information per grids summarized in Additional file 10). The Eastern Pyrenees harbored only haplotypes of lineage E, which appeared to be primarily distributed in this area. The Western Pyrenees harbored exclusively the haplotype 4. The five lineages were distributed across the Central Pyrenees. The majority of 10×10 km quadrats (54) harbored only one haplotype, although some contained two (8 quadrats) and three (3 quadrats) haplotypes. Moreover, few quadrats had haplotypes of two (grid coordinates I14, I25, J21, J22, K19) and three (J17, F8) lineages, and all of them fell into the Central Pyrenees.

Haplotype frequencies and molecular diversity indices per region are summarized in Tables 2 and 3. Central Pyrenees contained the highest genetic diversity (Hd = 0.789, π = 0.004). In this area haplotype 5 occurred in the highest percentage (32.85%), followed by H11 (21.30%) and H4 (20.22%). Eastern Pyrenees showed a lower value of genetic diversity in comparison with Central Pyrenees (Hd = 0.529, π = 0.0012). Only haplotypes of lineage E were found in the Eastern Pyrenees, with H11 showing the highest percentage (56.56%). The lowest molecular diversity was found in Western region, with H4 as the only haplotype.

**Table 2:** Number and frequency of each haplotype (%)

	**H2**	**H3**	**H4**	**H5**	**H6**	**H7**	**H8**	**H9**	**H10**	**H11**	**H12**	**H13**	**H14**
Eastern								39	10	69	4		
%								31.97	8.20	56.56	3.279		
Western			9										
%			100										
Central	6	1	56	91	16	1	1		18	59	4	2	22
%	2.17	0.36	20.22	32.85	5.78	0.36	0.36	0.00	6.50	21.30	1.44	0.72	7.94

**Table 3:** Genetic diversity in the three Pyrenean sections

**Pyrenean area**	***N***	***S***	***h***	***Hd***	***π***
Eastern	122	3	4	0.53	0.001
Western	9	0	1	0	0
Central	277	11	11	0.79	0.004

*N* = Number of sequences; *S* = Number of segregating sites; *h* = Number of haplotypes; *Hd* = Haplotype diversity; *π* = Nucleotide diversity.

F_ST_ values indicated that a phylogeographical pattern exists between the three regions (Table 4). The highest F_ST_ value (0.620) was found for genetic differentiation between Lineage E and the rest of lineages. AMOVA (analysis of molecular variance) analysis showed significant values for genetic differentiation among groups for the following two comparisons: (i) sampling locations partitioned according to Eastern, Western, and Central regions; and (ii) according to lineage E *versus* rest of samples. Nevertheless, variation values were higher among populations within groups (Table 5). The results of SAMOVA (spatial analysis of molecular variance) revealed two high F_CT_ values for clusters not including groups of a single population (Additional file 11). Variance among groups reached a value over 80% for the partition into seven geographic groups corresponding mainly to different plastid lineages and sublineages, without a clear geographic structure. The division into K = 5 clusters had the second highest variance among groups (78.77%). Both divisions clearly differentiated lineage E in a separate group. Such split largely corresponded to the biogeographic boundary for Eastern Pyrenees, which can be visualized in the distribution map of haplotypes (Figure 3). With K ranging between 8 and 20, F_CT_ values did not increase significantly and in most cases the newly defined groups comprised single populations. SAMOVA showed that a substantial portion of the cpDNA genetic variability was found among groups, whereas the differences among populations within groups accounted for less variation.The clustering BAPS (Bayesian Analysis of Population Structure) analysis resulted in a best partition of K = 8. These eight clusters primarily corresponded with the plastid lineages and sublineages (see Figure 1). In the admixture analysis, all individuals were unambiguously assigned to their respective group without any probability of being misplaced.

**Table 4:** **Pairwise F**_**ST**_**-values**

**Conventional F-Statistics from haplotype frequencies**			
	**Eastern**	**Central**	**Western**
Eastern	0.000		
Central	0.218*	0.000	
Western	0.588*	0.318*	0.000
	Lineage E	Rest	
Lineage E	0.000		
Rest	0.620*	0.000	

Comparisons were performed among populations from the Eastern, Central and Western Pyrenees, and among Lineage E and the remaining lineages. Asterisk indicates statistical significance (p < 0.05).

**Table 5:** Results of AMOVA

**Grouping hypothesis**	**Va (percentage)**	**Vb (percentage)**	**Vc (percentage)**	**P**
Eastern-Central-Western	0.10 (22.67%)	0.33 (71.01%)	0.03 (6.32%)	0.00
Lineage E-rest of lineages	0.19 (38.01%)	0.28 (56.10%)	0.03 (5.88%)	0.00

Va: variation among groups; Vb: variation among populations within groups; Vc: variation within populations.

### Demographic history

**Figure 4:**
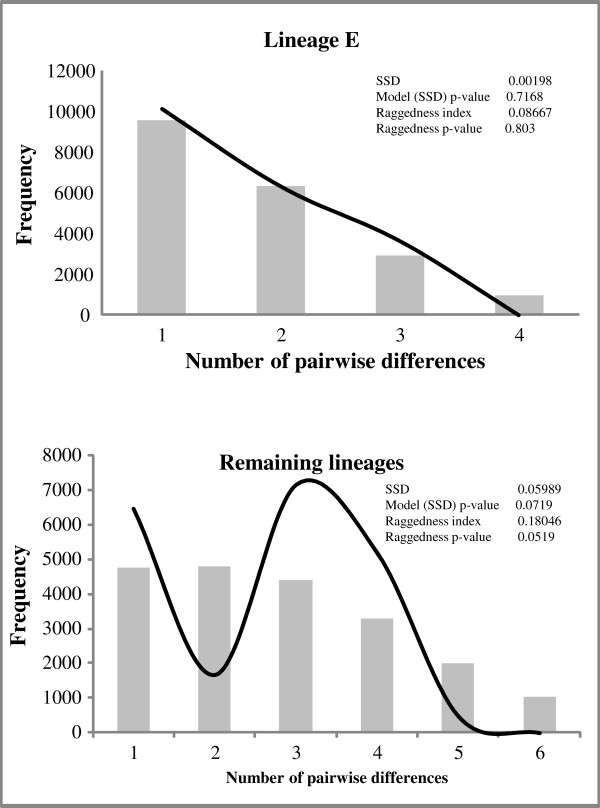
**Results of mismatch distribution analyses.** Pictures in right are results of mismatch distribution. Goodness of fit of the observed vs. the theoretical mismatch distributions under a sudden expansion model was tested using the sum of squared deviation (SSD) and raggedness index. Not significant P > 0.10.

Neither Tajima’s D nor Fu’s FS was significantly different from zero for lineage E (Table 6). Nevertheless, the mismatch distribution analyses did not reject the sudden expansion model for this lineage. The unimodal peak and nonsignificant Harpending’s raggedness index (Figure 4) are indicative of recent demographic expansion [29]. For the group of the remaining lineages, Tajima’s D and Fu’s Fs were negative but not significant. Mismatch distribution analyses rejected the range expansion hypothesis for this group by a bimodal distribution and significant SSD (sum of square deviations) and RAG (Harpending’s raggedness index) values (Figure 4).

**Table 6:** Tajima’s D and Fu’s FS test

	**Tajima’s D**	**Tajima’s D p-value**	**Fu’s FS**	**FS p-value**
Lineage E	1.35	0.89	1.95	0.82
Remaining lineages	−0.14	0.50	−0.84	0.43

Signification level P < 0.10.

## Discussion

### Origins of *Antirrhinum* in the Pyrenees

Regarding the geographic origin of the Pyrenean lineages, an important historical connection between the northeast Iberian Peninsula and the Pyrenees is well supported (Figure 2, Additional file 6). It is notable that none of the analyses identified evolutionary relationships between the lineages from the Pyrenees and those from the South-western Alps, which is a connection found in other plant species with similar distributions *e.g. Erodium*, [30], *Primula*, [31]. The lack of a connection between these two areas is also supported by nrITS sequences (Liberal et al. unpublished, see additional file 12). This is a rather unexpected, but essential, result since the yellow-flowered populations that inhabit the Alps and the Pyrenees have recently been circumscribed into a single species (*A. latifolium* Mill.) by some taxonomists [32,33]. The phylogenetic and dating analyses provide strong support for the divergence of Pyrenean sublineages in the Quaternary but poor support for sister-group relationships at the most basal nodes. The analyses presented here and those of Vargas et al. [22] support the first hypothesis herein posit that the Pyrenean lineages are the result of long-standing isolation and geographic differentiation from a widespread ancestor in the Pleistocene. However, additional nuclear data, such as single-copy nuclear markers, would be required to determine whether the unresolved basal relationships are attributable to hard or soft polytomies [34].

### East–west geographical structure of the genetic diversity

The main genetic discontinuity identified by the analyses (SAMOVA, AMOVA, and FST) indicated the differentiation of the Lineage E from the remaining lineages. These two groups basically correspond to the division between the Eastern and Central-West Pyrenees (Figure 3). This cryptic transition area, which approximately follows the abrupt valley of the Segre River, is supported by the phylogeographic gap in the southeastern part of the Pyrenees that was already identified in previous studies of animals and plants *e.g.*[35-38]. A similar phytogeographic boundary was previously proposed based on species distributions in the Pyrenees [39,40]. The high level of differentiation between the Eastern Pyrenean lineage (Lineage E) and the remaining lineages, suggests the isolation of eastern populations over more than one glacial cycle.

Interestingly, the eastern and central areas may have experienced different historical processes during the last glaciations. According to the results of the mismatch distribution analyses, the observed genetic structure in the Eastern Pyrenees is congruent with a range expansion process, whereas this pattern was not observed in the Central Pyrenees. By contrast, the strong evidence for the genetic clustering of Central Pyrenean samples, which was detected by the BAPS and SAMOVA analyses, as well as the significant genetic differentiation among the different lineages that occur in the area, support long-standing genetic isolation in different local refugia without an extensive interglacial (or postglacial) range expansion as already inferred for *Pinus uncinata* [according to [19]. However, the homogeneously distributed genetic diversity indicates that moderate gene flow occurred among Central Pyrenean refugia during interglacial periods. Thus, two different historical processes were inferred: (i) limited extinction/expansion during glacial cycles in the central area, and (ii) population expansion (probably pre-dated by a demographic bottleneck based on the reduced number of lineages and haplotypes) in the eastern area, may explain the observed differences in the genetic structure and diversity of both regions. A similar interpretation was proposed for *Ramonda myconi* to explain genetic differences between populations from Central and Eastern Pyrenees [41] where significant evidence was presented to support the existence of an important refuge near the central area. Further support for a large refuge in Central Pyrenees was also found by palynological data [42,43], the patterns of phylogeographic concordance among different species [44], and the high diversity of habitats, species richness, and endemic species [63 of the 160 endemic plant species of the Pyrenean flora are exclusively from the Central Pyrenees; [45]. In agreement with this and other studies *e.g.*[46], a scenario of multiple refugia in mountains adjacent to the Central Pyrenees (*i.e.*, the southern mountains of the pre-Pyrenees) is gaining more support (see Figure 3).

Similar to the pattern found in *Antirrhinum*, *Ramonda myconi* also exhibits considerable differentiation but reduced genetic diversity in the eastern population [41]. Fossil pollen records and molecular studies suggest the presence of smaller refugia in the southeastern Pyrenees, along the Mediterranean coast [47-49]. Recent studies indicate that climatic fluctuations during the last glacial period affected the Eastern Pyrenees (Mediterranean coast) more strongly than the Central Pyrenees, at least during the last 50,000 years. Overall, more pronounced oscillations between arid and humid periods prevailed in the easternmost regions [50,51]. Thus, severe reductions in population sizes and the isolation of peripheral populations at the edge of the distribution might have led to genetic drift and local adaptations via selective forces see (see [52-55]). Future comparative phylogeographic studies and coalescent demographic analyses could help to elucidate the historical processes responsible for the phylogeographic and floristic configuration of the Pyrenees.

### Migration routes across the Pyrenees

The colonisation of the Western Pyrenees occurred from the Central Pyrenees. Indeed, the western populations have a low level of genetic diversity, which is shared by central populations. Such gradual genetic impoverishment observed from the Central to the Western Pyrenees is interpreted as a recent colonisation, which originated in the most central parts of the mountain range see [4,5,56,57]. The results of the DPA analysis also indicate that the postglacial colonisation of the southern French basin occurred primarily from the Eastern Pyrenees (Figure 3). The geographic distribution of the haplotypes and the mismatch distribution analyses (Figure 4) suggest a gradual colonisation of southern France from northeastern Iberia, where the edge of the glacial distribution range may have retreated early see [58].

### Similarities between the Pyrenees and the Alps

The causes of the separation of *Antirrhinum* lineages and plant species by the abrupt valley in the Pyrenees (Segre River) are unclear. Similar west–east vicariant lineages abutting along the Etsch Valley and the Brenner Pass in the Alps have been reported [18,59-61]. Indeed, this discontinuity accounts for the most important phytogeographic and biogeographic boundary in the Alps *e.g.*[62-64]. Similar genetic and floristic discontinuities are found in the Alps and the Pyrenees, which suggest that plant extinctions due to Holocene glaciers and further recolonisation events were not distributed homogeneously across each of the two mountain ranges. The major effect of the glacial history of mountain valleys in the current configuration of intra- and inter-specific genetic diversity requires further investigation.

The present study also underlines the importance of periglacial areas for the survival of species during glacial periods of the Quaternary. The pre-Pyrenees range, is recognised as an important glacial refuge where many animal and plant species survived during cold episodes [30,38,65,66]. Previous studies focused on the Alps have also recognised the importance of marginal and peripheral areas at lower altitudes with warmer climates as refugia for mountain species, where their present-day distribution were almost entirely glaciated reviewed in [18].

## Conclusions

Our results do not support connections between the *Antirrhinum* populations of the Alps and those of the Pyrenees. Instead, the analyses agree with ancient connection with Iberia followed by persistence of *Antirrhinum* populations in the Pyrenees, at least since Late Pleistocene times. The three Pyrenean sections (Western, Eastern, and Central) have different colonisation histories. The Western Pyrenean populations are closely related to the central populations, which is here interpreted as evidence for a recent westward colonisation (see Figure 2). It is likely that the Central pre-Pyrenees played an important role in the maintenance of the genetic diversity of *Antirrhinum* by providing suitable conditions for the establishment of probably separate refugial populations during glacial periods. A significant influx of migrants from adjacent refugia (Central pre-Pyrenees) during the interglacial and postglacial periods would account for the genetic diversity found in the Central Pyrenees. However, the current genetic structure of *Antirrhinum* in the Eastern Pyrenees appears to have been shaped by range expansion, which was probably preceded by range contraction at the southernmost edge of this area, from where little gene flow may have occurred into the Central Pyrenees over several glacial cycles. According to this, the Eastern pre-Pyrenees also appear to have been a refugial area, although less important than the central pre-Pyrenees, from where *Antirrhinum* colonisation southern France.

## Methods

### Study species

Maternally inherited plastid markers often display stronger differentiation between populations than biparental markers due to their smaller effective size and lack of recombination [56,67-69]. Because of this, reconstruction of migration histories by means of seed dispersal have been traditionally based on organelle markers once maternal inheritance has been documented [70,71]. To this end, artificial crossing experiments under greenhouse conditions were conducted between four *Antirrhinum* taxa (*A. mollissimum*, *A. majus* subsp. *majus*, *A. controversum* and *A. charidemi*) (Additional file 13).

### Sampling strategy and DNA sequencing

We collected a total of 452 individuals from 99 populations of *A. molle*, *A. sempervirens*, *A. majus* subsp. *majus* and *A. majus* subsp*. striatum* from the Pyrenees and adjacent mountains. In addition, a total of five individuals from five populations of *A. latifolium* distributed across the Province/southern Maritime Alps (Additional file 3) were analyzed given that this species has historically been considered closely related to *A. majus* subsp*. striatum*[32,33]. The number of populations sampled per species depended on their distribution range: *A. molle* (3), *A. sempervirens* (9), *A. majus* subsp. *majus* (61) and *A. majus* subsp*. striatum* (26) respectively. Particular effort was made to achieve a sampling as complete as possible for the entire mountain range (see Figure 3). All individuals were collected in the field and dried in silica gel. Total genomic DNA was extracted using Dneasy Plant Mini Kit (QIAGEN Inc., California).

Two sequencing strategies were adopted. First, *trn*K-*mat*K and *trn*S-*trn*G intergenic spacer sequences were obtained for one individual from each population, and added on to the sequence data matrix from Vargas et al. [22]. This dataset (hereafter *Antirrhinum matrix*) was used to infer the colonisation history of Pyrenean lineages by phylogenetic and phylogeographic analyses. Second, *trn*S-*trn*G intergenic spacer sequences were obtained for the 452 individuals (1-6 individuals per population) from the Pyrenees. This sequence matrix (hereafter *Pyrenees matrix*) was used to investigate underlying spatial genetic structure and genetic diversity across the Pyrenees.

The *trn*K-*mat*K and *trn*S-*trn*G regions were amplified as in Vargas et al. [22]. PCR products were outsourced for sequencing to a contract sequencing facility (Macrogen, Seoul, South Korea) on an ABI Prism® 3730xi DNA sequencer, using the same primer sets as for PCR. Resulting sequence data were assembled and edited using Geneious Pro v5 [72]. Both datasets were aligned with the MAFFT v.6.814b alignment tool [73], as implemented in Geneious Pro with default parameters. Further adjustments were made by visual inspection.

### Phylogenetic analyses (Antirrhinum matrix)

Bayesian phylogenetic analyses were performed on the two separate matrices (*trn*S-*trn*G/*trn*K-*mat*K) to examine plastid gene tree congruence. *Gambelia speciosa* and *Misopates orontium* were selected as the outgroup based on previous phylogenetic evidence [20]. In addition, phylogenetic analyses for 190 *trn*S-*trn*G/*trn*K-*mat*K concatenated sequences were conducted using Bayesian inference (BI), as implemented in MrBayes v3.1.2 [74], maximum likelihood (ML), as implemented in PhyML 3.0 [75], and maximum parsimony (MP), as implemented in TNT 1.1 [76] (see Additional file 14 for details).

### Estimation of divergence times (Antirrhinum matrix)

A relaxed molecular-clock approach implemented in BEAST v.1.6.2 [77] was used to estimate the divergence times between *Antirrhinum* Pyrenean lineages. This software estimates the phylogenetic tree and node ages simultaneously. Since no reliable fossils of *Antirrhinum* are known to date, we implemented a secondary basal calibration following [78] but with some modifications. The divergence time between the basalmost *Antirrhinum* lineages (crown node) was modelled as a normal distribution with mean = 4.5 Ma and standard deviation = 1.8, on the basis of a previous analysis of *ndh*F sequences of Antirrhineae that included two most distant *Antirrhinum* species (*A. meonantum* and *A. majus* subsp. *majus*, following results of phylogenetic analyses). The latter analysis incorporated a calibration of 74 Ma for the divergence time between Oleaceae and Antirrhineae [79], and minimum stem-age constraints for Lamiales families and tribes based on five fossils see [80] for details on fossils].

### Ancestral area reconstructions (Antirrhinum matrix)

A discrete phylogeographic analysis (DPA) implemented in BEAST [81] was performed to assess the probability distribution of the geographic locations in each node. A total of 14 discrete areas were delimited: (i) the four Iberian quadrants (northeastern Iberia, NE; northwestern Iberia, NW; southeastern Iberia, SE; southwestern Iberia, SW), as divided by the geographical coordinates 40ºN/5ºW see [22]; (ii) Eastern, Central and Western Pyrenees, as the three recognized biogeographic regions within the Pyrenees (see below); (iii) the other two northern areas sampled nearby the Pyrenees (Southern French basin and South-western Alps); and (iv) the remaining five areas sampled across Mediterranean basin (northern Africa, Sicily, Sardinia, Italian Peninsula and Turkey). Statistical significance for the rates of the dispersal events was assessed via Bayes factor (BF) test as described by Lemey et al. [81]; see Additional file 14 for details].

Additional ancestral range reconstructions were conducted using the Bayesian time-calibrated molecular phylogeny with the aim to discriminate between northern and southern origin of Pyrenean lineages. For this purpose, only four areas were delimited (i) Iberian Peninsula, (ii) Pyrenees and adjacent areas, (iii) South-western Alps, and (iv) samples from the Mediterranean basin (excluding Iberia). Ancestors were allowed to be present in all of them. Distribution ranges of sequences (haplotypes) instead of species was used see [80]. Two alternative reconstruction methods were used: (a) Statistical Dispersal-Vicariance Analysis (S-DIVA) implemented in the program RASP 1.1 [82], and (b) dispersal-extinction-cladogenesis analysis (DEC) implemented in the software package Lagrange v2.0.1 [83]; see Additional file 14 for details].

### Haplotype network analyses (Antirrhinum and Pyrenees matrices)

Haplotype networks using the *Antirrhinum* and *Pyrenees* matrices (hereafter *Antirrhinum* and *Pyrenees networks,* respectively) were constructed under the statistical parsimony framework in TCS 1.2.1 [84]. The maximum number of differences among haplotypes, as a result of single substitutions, was calculated with 95% confidence limits. Indels of varying lengths resulting from the expansion/contraction of polyA and polyT were excluded due to their high levels of homoplasy [85]. One indel of 9 bp was found in several samples of the population *Villefranche-de-Conflent* of *A. majus. subsp. striatum* for the *trn*S-*trn*G plastid region (Additional file 3) and was re-coded as a single character and treated as a fifth character state in. For the *Pyrenees network*, one sequence of *A. braun-blanquetii* (EU673480) was used as the outgroup, since this sequence appears to retain the ancestral haplotype in the *Antirrhinum network* analysis.

### Genetic diversity and geographic structure (Pyrenees matrix)

An analysis of genetic diversity was carried out across the three recognized biogeographic regions in which the Pyrenees range is divided (Eastern, Central and Western Pyrenees) (see Figure 3a). The boundaries of these three biogeographic areas, although with slight differences, have been traditionally established by both geologists [86,87] and phytogeographers [39,40,88-91] on the basis of geologic, climatic and floristic data. The boundaries of these areas are approximately defined by the Gallego River, between Central and Western Pyrenees, and the Segre River, between Central and Eastern Pyrenees. Haplotype frequencies and molecular diversity indices for each biogeographic area were calculated using DnaSP v5 [92]. In addition, to identify potential hotspots of genetic diversity across the Pyrenees, individuals were geographically grouped by means of a 10×10 km grid. Charts representing haplotype frequencies were constructed for each grid cell, which was named by a generic letter–number code (Figure 3).

To identify genetic subdivisions among the Eastern, Central and Western Pyrenees, we performed an analysis of molecular variance (AMOVA) [93]. Pairwise F_ST_ statistics were also calculated to estimate genetic distances. Both analyses were performed by using ARLEQUIN [94]. Additionally, an AMOVA was performed in order to assess the partitioning of variance between the Lineage E (primarily distributed in the eastern part of the Pyrenees) and the rest of lineages (see below). Furthermore, to evaluate the optimal grouping of the sampled sites without *a priori* assumptions, a Bayesian analysis of population structure, implemented in the BAPS software, version 5.3 [95,96], and a spatial analysis of molecular variance (SAMOVA) implemented in the software package SAMOVA 1.0 [97] were also performed (see Additional file 13 for details on analyses).

### Demographic history (Pyrenees matrix)

Past range expansions were assessed for the two partitioned population groups (lineage E and remaining populations, see below) using two methods. First, we tested for range expansion using Tajima’s D [98] and Fu’s Fs statistics [99] which, assuming neutrality, are expected to be significantly negative under population expansion. Significance for each test was assessed by generating null distributions from 10000 simulations. Second, we also conducted a mismatch analysis of cpDNA sequence differences, comparing the observed frequencies of pairwise differences of haplotypes with those expected under a sudden expansion (pure demographic expansion) model [29,100]. Under expansion, the distribution of the observed differences is expected to be unimodal, whereas, under population contraction or genetic subdivision, a multimodal distribution is expected. Statistical significance was determined by 10000 bootstrap replicates. Goodness of fit was assessed by the sum of square deviations (SSD) and the Harpending’s raggedness index [101] between the observed and the expected mismatch. These analyses were conducted using ARLEQUIN 3.5. [102].

## Competing interests

The authors declare that they have no competing interests.

## Authors’ contributions

IML generated the molecular data, performed the analyses and drafted the manuscript. PV conceived of the study, and participated in its design and coordination. Both authors participated in the interpretation of the data and co-wrote the manuscript. MB and CS contributes to the generation of molecular data. CT & MB contributed ideas and suggestions for improving the final manuscript. All authors participated in the obtaining of plant material. All authors read and approved the final manuscript.

## Supplementary Material

Additional file 1 Comparison of the different taxonomic treatments of two studied taxa according to different authors. Click here for file

Additional file 2 Phylogeographical patterns of alpine and montane angiosperms distributed in the Pyrenees. Review based on geographical origin and population dynamics of species during the Quaternary. Click here for file

Additional file 3 **Samples used for trnS-trnG and trnK-matK sequencing.** Population numbers after species names in brackets. IML, Isabel Liberal’s collection numbers; EDB, Laboratoire Evolution et Diversité Biologique. For others Voucher see Vargas et al. [22]. Asterisks (*) after GenBank accession numbers refer to those from Vargas et al. [22]. Nomenclature from Vargas et al. [22] is followed except for A. controversum (=A. barrelieri). The samples here named A. majus subsp. striatum (populations 1-3) were treated like A. latifolium in Vargas et al. [22]. Abbreviations used: DEC = dispersal-extinction-cladogenesis analysis; S-DIVAS = Statistical Dispersal-Vicariance Analysis; DPA = Discrete Phylogeographic Analysis; SE = South-east; SW = South-west; NE = North-east; NW = North-west; Pyr. = Pyrenees; Pre-Pyr. = Pre-Pyrenees; Pyr. & adj. = Pyrenees and adjacent areas; S. French basin = Southern French basin; E. Pyrenees = Eastern Pyrenees; C. Pyrenees = Central Pyrenees; W. Pyrenees = Western Pyrenees. Click here for file

Additional file 4 Bayesian phylogenetic tree constructed with plastid *trnS-trnG* sequences *(Antirrhinum matrix).* Click here for file

Additional file 5 Bayesian phylogenetic tree constructed with plastid *trnK-matK* sequences (*Antirrhinum matrix).* Click here for file

Additional file 6 **Results of the Bayes Factor (BF) test for *Antirrhinum matrix.*** Are indicated the Bayes Factors with well-supported rates of dispersal (values of BF > 3). Click here for file

Additional file 7 **Ancestral area reconstruction (*Antirrhinum matrix)* using DIVA method as implemented in the software RASP (ex S-DIVA; Yu et al. (2010)).** At each node, the most likely inferred ancestral areas are drawn. The four areas are as follow: Iberian Peninsula (A), Pyrenees (B) Mediterranean Basin (C) and South-western Alps (D). Vicariant event among Alps (D) and the Iberian Peninsula (A) is indicated by * and node support. Click here for file

Additional file 8 **DEC (Lagrange) Inference (*Antirrhinum matrix).*** The optimal area reconstruction on the branch is represented by a two-letter code (*i.e.* A|A), being the area on the left the one inherited by the upper daughter branch, and the area on the right the one inherited by the lower daughter branch. Click here for file

Additional file 9 **Haplotype network of the *Antirrhinum matrix.*** For geographic abbreviations see [22]. Click here for file

Additional file 10 **(correspondence with Figure** 3**).** Distribution of the lineages and haplotypes on the grid (*Pyrenees matrix*). For each taxon, the first column indicate the number of the haplotypes and number of samples showing that haplotype (in brackets), while the second column indicates the lineage corresponding to each haplotype. The cells shaded in grey show areas harboring at least two lineages. Click here for file

Additional file 11 Results of SAMOVA analyses (*Pyrenees matrix).* Click here for file

Additional file 12 **Bayesian phylogenetic tree constructed with nrITS sequences published in **22**plus the five new nrITS sequences corresponding to the samples collected across South-western Alps for this study.** Click here for file

Additional file 13 **Haplotypes indentified in the interspecific crossing study.** Abbreviations used: maj = *A. majus*; con = *A. controversum*; cha = *A. charidemi*; moll = A*. mollissimum*. Click here for file

Additional file 14 Methods. Click here for file

## Data Accessibility

Genbank Accession numbers for DNA sequences: *trn*S-*trn*G (KJ911400-KJ911861) and *trn*K-*mat*K (KJ911294-KJ911399).
