# Supplementary material for: The evolutionary history of Antirrhinum in the Pyrenees inferred from phylogeographic analyses
Source: BMC Evol Biol. 2014 Jun 26;14:146. doi: 10.1186/1471-2148-14-146 (PMC4099501; doi:10.1186/1471-2148-14-146)
Supplement: Additional file 2 — Phylogeographical patterns of alpine and montane angiosperms distributed in the Pyrenees. Review based on geographical origin and population dynamics of species during the Quaternary. [file 1471-2148-14-146-S2.docx]

| **Additional file 2**. **Phylogeographical patterns of alpine and montane angiosperms** **distributed in the Pyrenees**. Review based on geographical origin and population dynamics of species during the Quaternary. | | | | |
| --- | --- | --- | --- | --- |
| **Species** | **Habitat& ecological requirements** | **Phylogeographic pattern** | **Area genetically connected with the Pyrenees** | **References** |
| *Hippophaë rhamnoides* L. (Elaeagnaceae) | Cold-tolerant shrub occurring in moderate-low altitudes | Two recent expansion events from Alps to the Pyrenees. | North | [[102](#_ENREF_102)] |
| *Saxifraga oppositifolia* (Saxifragaceae) | Artic-alpine | Close relationship between populations from the Alps and the Pyrenees | North | [[103](#_ENREF_103),  [104](#_ENREF_104)] |
| *Gentiana nivalis* L. (Gentianaceae) | Artic-alpine | Colonization of the Pyrenees from Central Europe | North | [[105](#_ENREF_105)] |
| *Saxifraga paniculata* (Saxifragaceae) | Artic-alpine | Colonization of the Pyrenees from the Alps | North | [[106](#_ENREF_106)] |
| Salix herbacea L. (Salicaceae) | Artic-alpine | Ancient colonization of the Pyrenees from the Alps | North | [109] |
| *Pulsatilla vernalis* (L.) Mill. (Ranunculaceae) | Alpine | Colonization of the Pyrenees from the Alps | North | [[107](#_ENREF_107)] |
| *Carex curvula* ssp. *curvula* (Cyperaceae) | Alpine | Recent colonization of the Pyrenees from the Alps. | North | [[108](#_ENREF_108)] |
| *Androsace vitaliana* (Primulaceae) | Alpine | Colonization of the Pyrenees from the Alps | North | [[110](#_ENREF_110)] |
| Arenaria tetraquetra L. (Caryophyllaceae) | Alpine | Colonization of the Pyrenees from SE Iberia. | South | [103] |
| *Ranunculus glacialis*  L. (Ranunculaceae) | Artic-alpine | Populations more likely to have persisted over long periods | Allopatric differentiation/ long-term persistence | [[111](#_ENREF_111)] |
| *Trollius europaeus*  L. (Ranunculaceae) | Artic-alpine | Alpine and Pyrenean populations represent moderately fragmented relics of large southern ancestral populations | Allopatric differentiation/ long-term persistence | [[112](#_ENREF_112)] |
