# Supplementary figures and images for: The evolutionary history of Antirrhinum in the Pyrenees inferred from phylogeographic analyses
Source: BMC Evol Biol. 2014 Jun 26;14:146. doi: 10.1186/1471-2148-14-146 (PMC4099501; doi:10.1186/1471-2148-14-146)

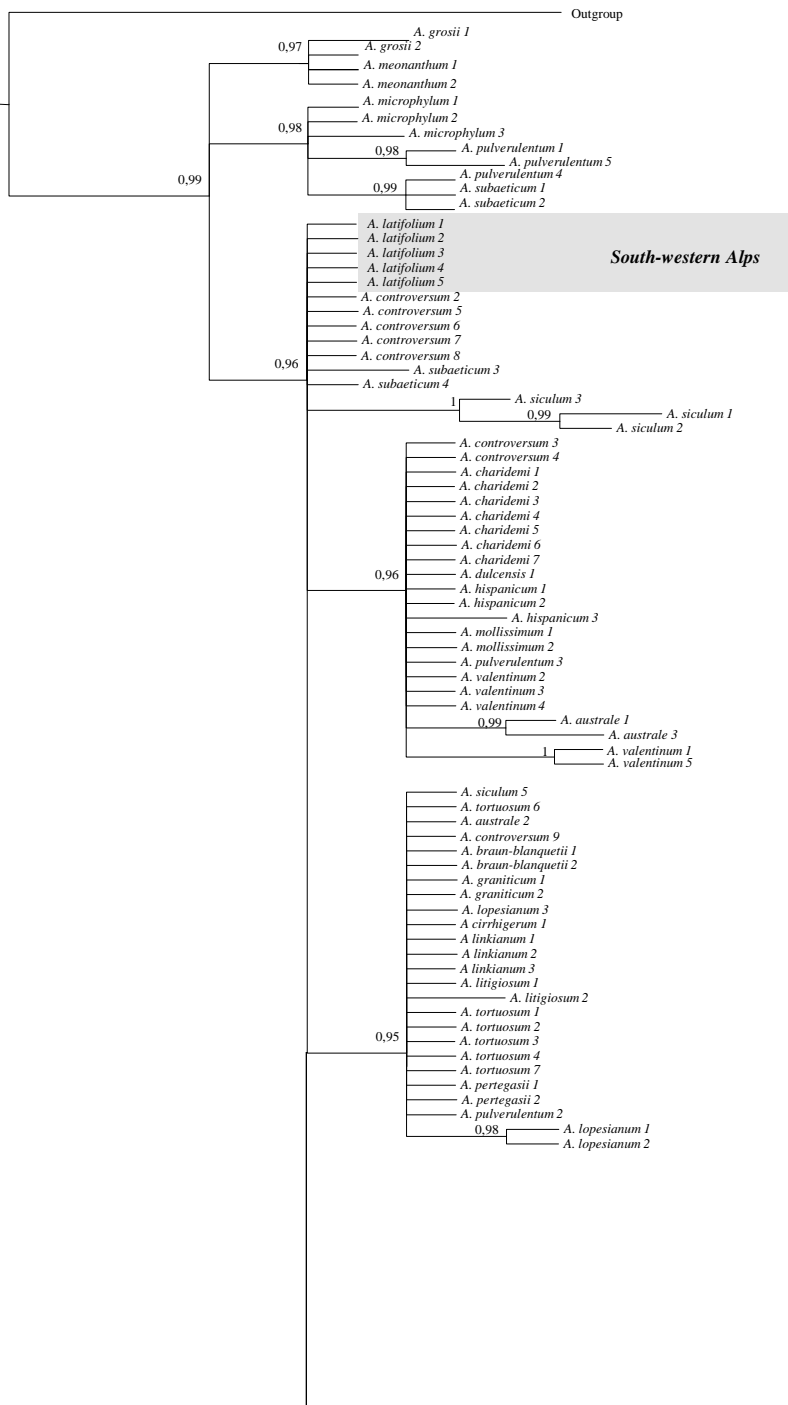

*trnS-trnG*

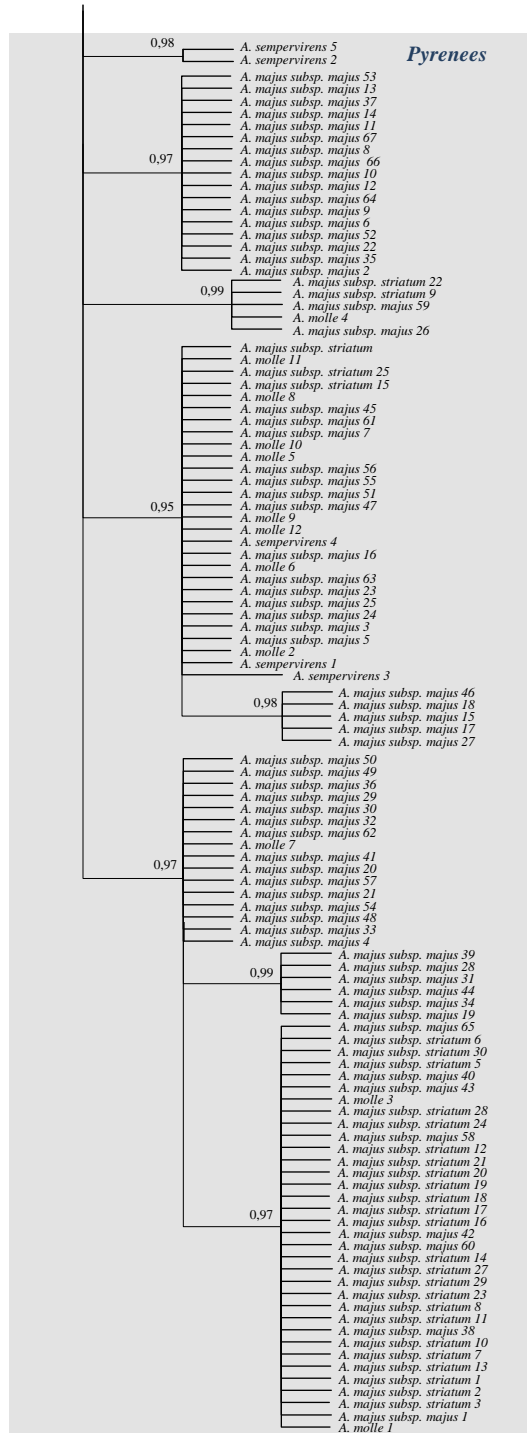

Supplement: Additional file 4 — Bayesian phylogenetic tree constructed with plastid trnS-trnG sequences (Antirrhinum matrix). [file 1471-2148-14-146-S4.pdf]

Outgroup

trnK-matK

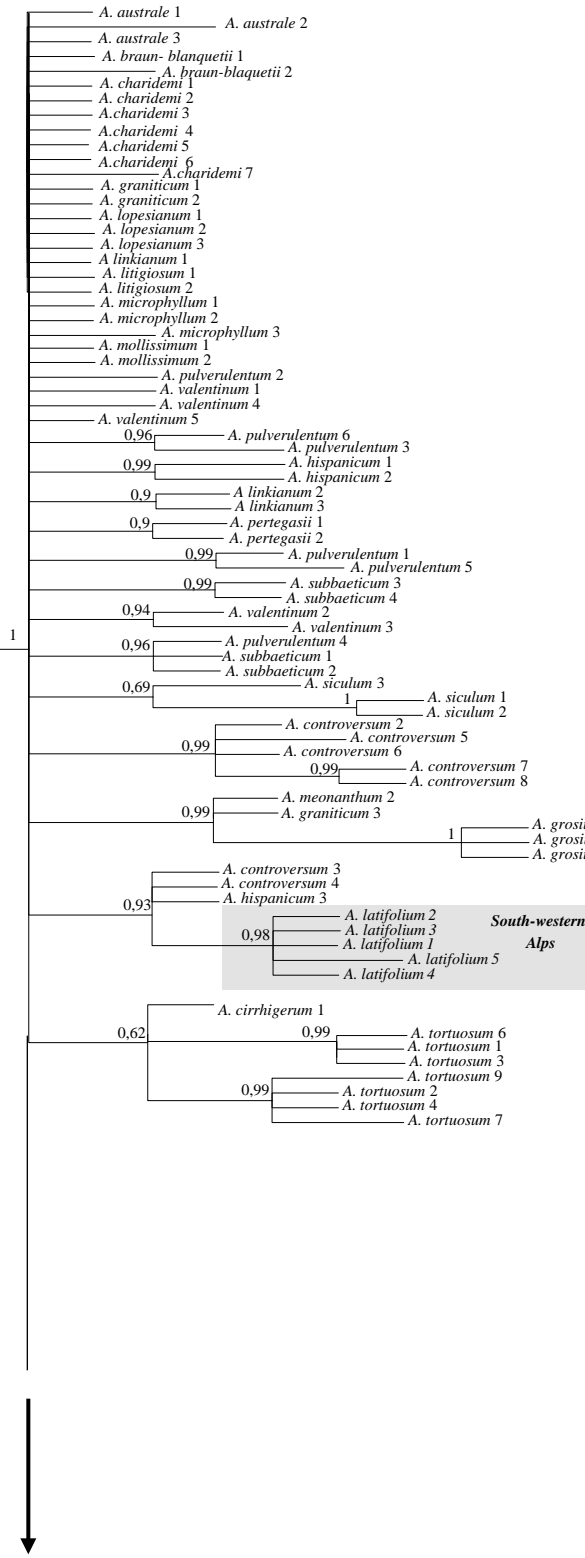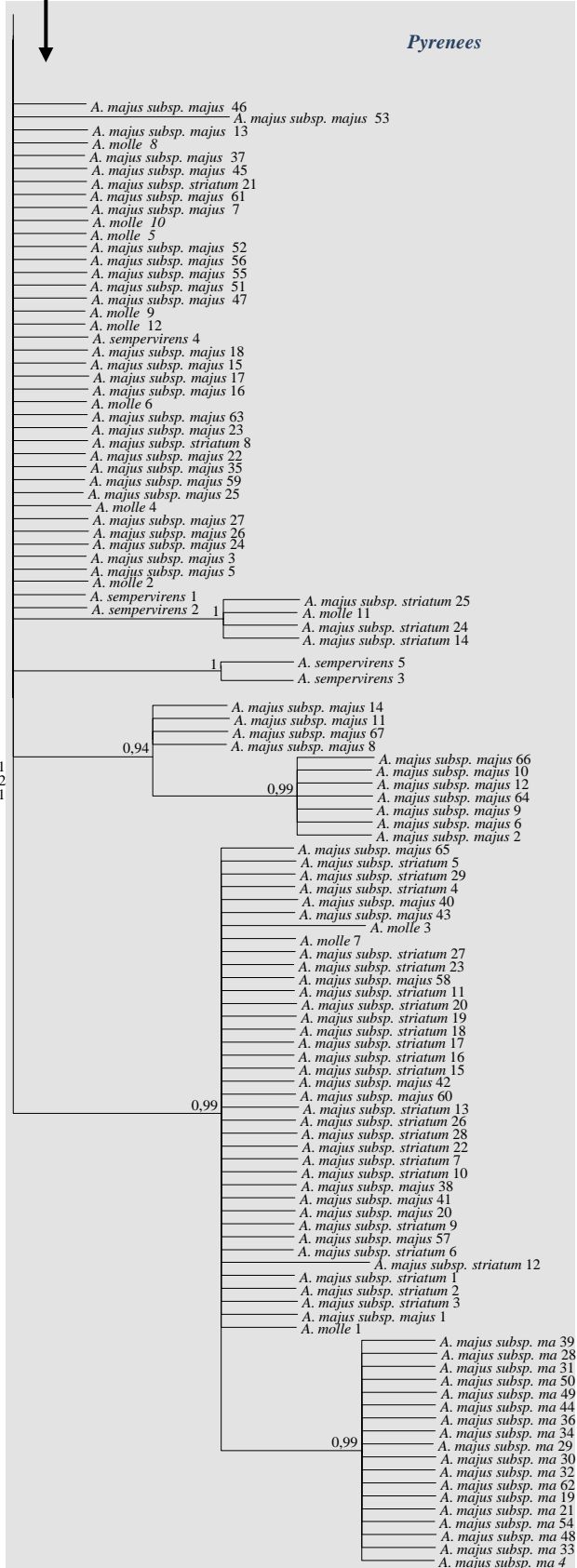

Supplement: Additional file 5 — Bayesian phylogenetic tree constructed with plastid trnK-matK sequences (Antirrhinum matrix). [file 1471-2148-14-146-S5.pdf]

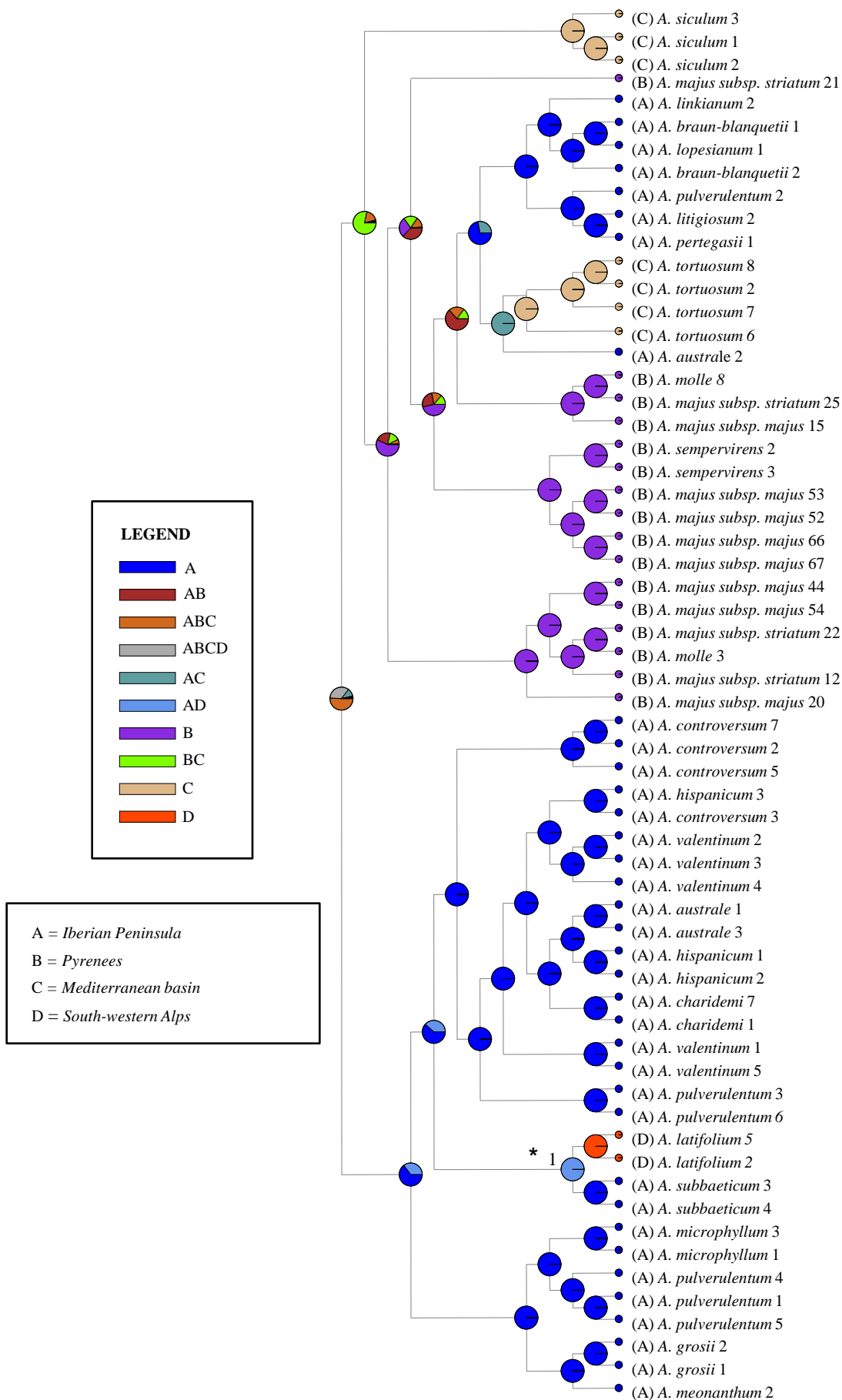

Supplement: Additional file 7 — Ancestral area reconstruction (Antirrhinum matrix) using DIVA method as implemented in the software RASP (ex S-DIVA; Yu et al. (2010)). At each node, the most likely inferred ancestral areas are drawn. The four areas are as follow: Iberian Peninsula (A), Pyrenees (B) Mediterranean Basin (C) and South-western Alps (D). Vicariant event among Alps (D) and the Iberian Peninsula (A) is indicated by * and node support. [file 1471-2148-14-146-S7.pdf]

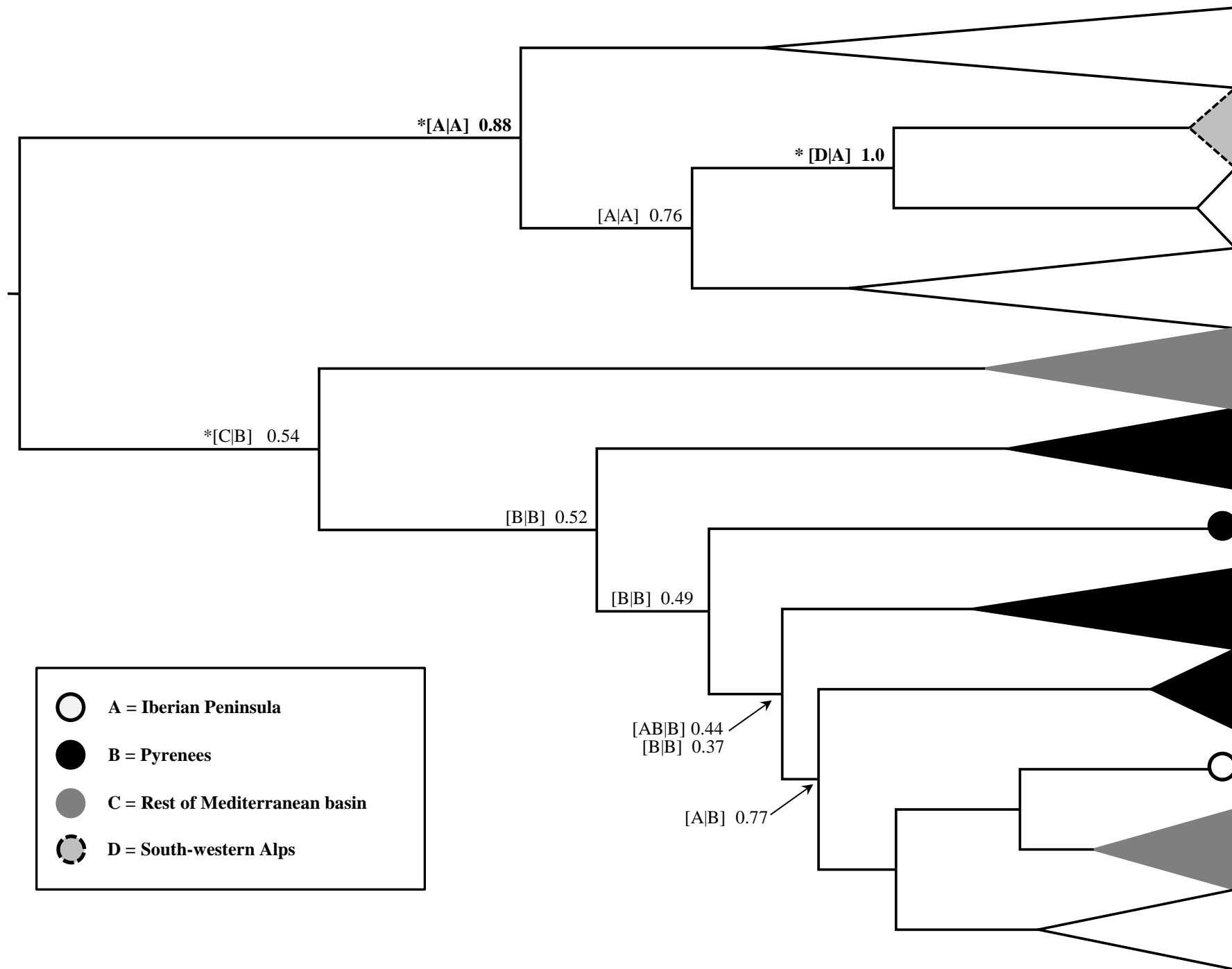

Supplement: Additional file 8 — DEC (Lagrange) Inference (Antirrhinum matrix). The optimal area reconstruction on the branch is represented by a two-letter code (i.e. A|A), being the area on the left the one inherited by the upper daughter branch, and the area on the right the one inherited by the lower daughter branch. [file 1471-2148-14-146-S8.pdf]

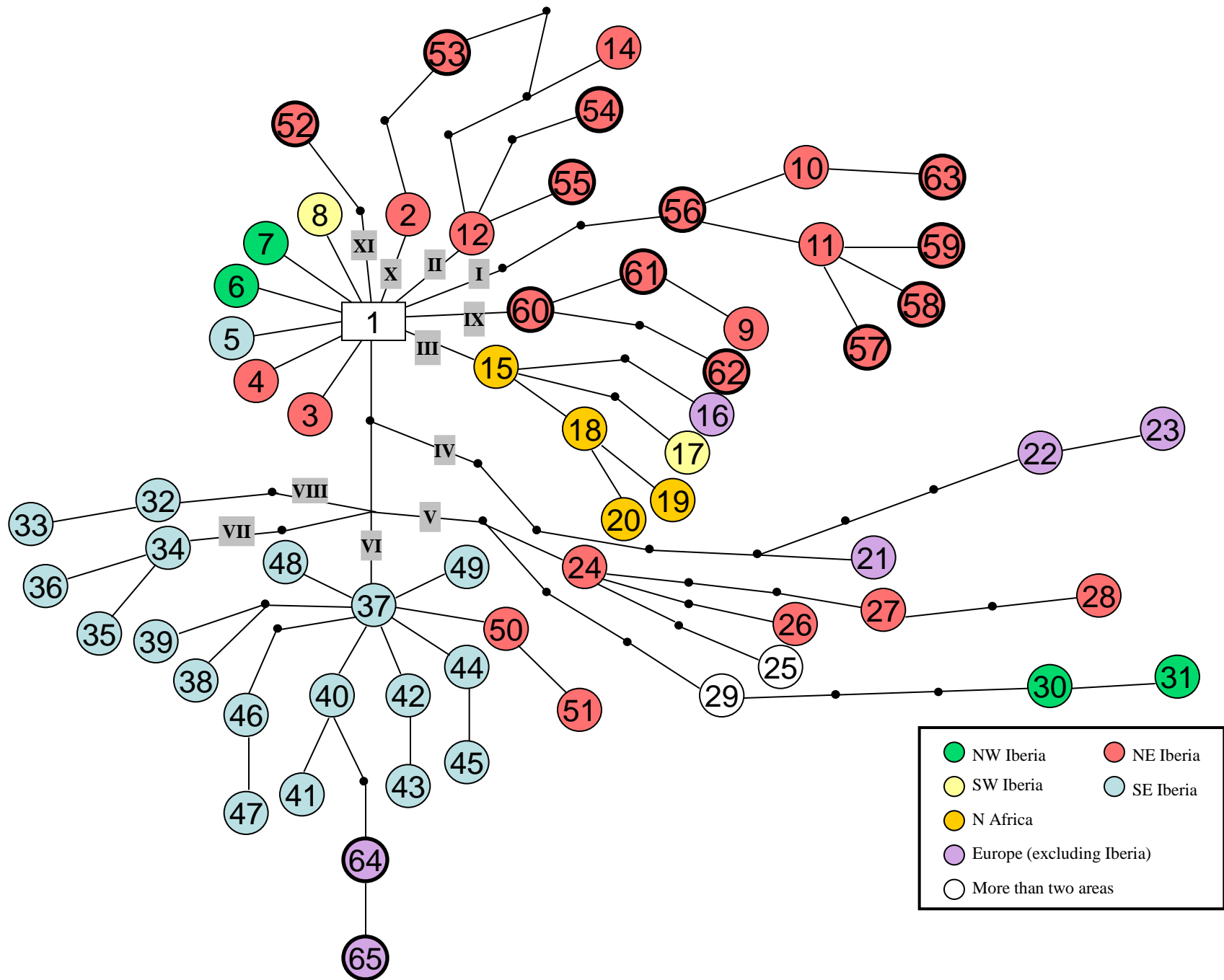

Supplement: Additional file 9 — Haplotype network of the Antirrhinum matrix. For geographic abbreviations see [22]. [file 1471-2148-14-146-S9.pdf]

### SAMOVA

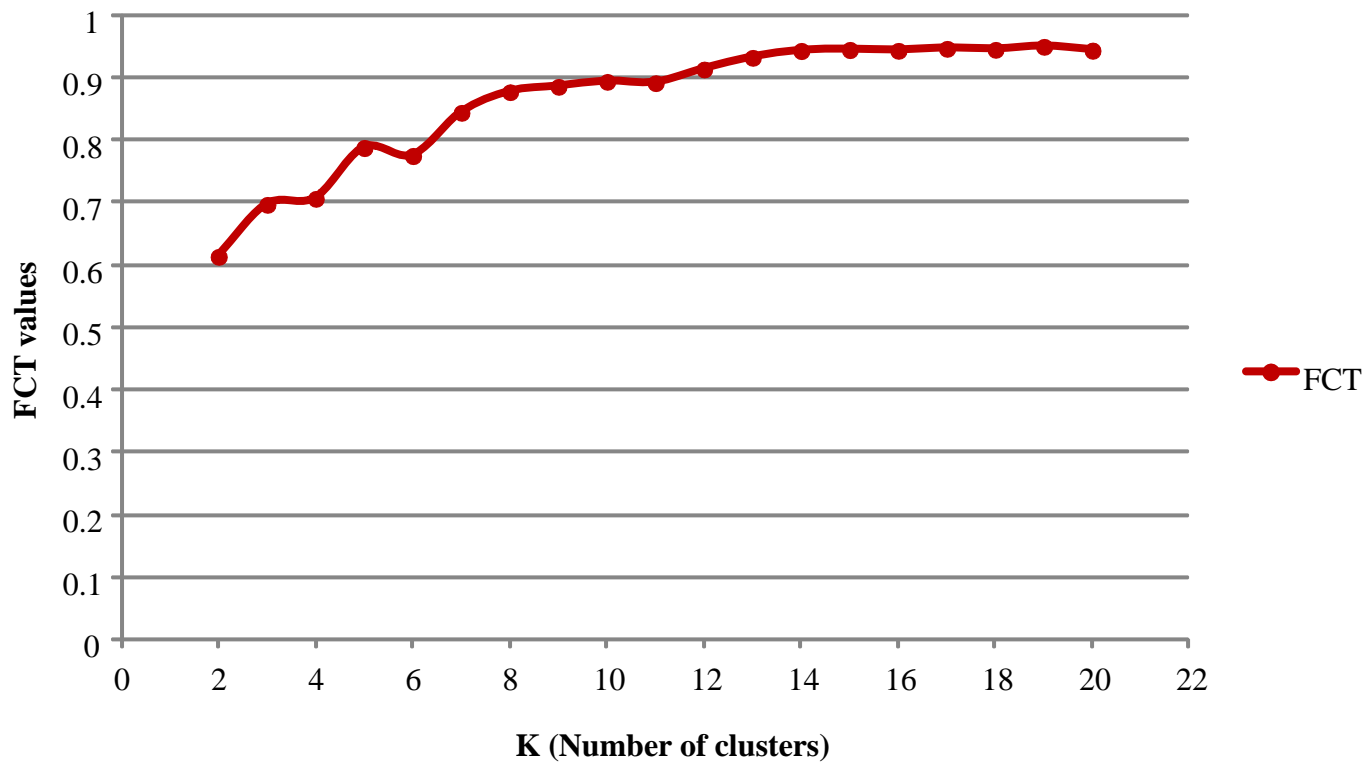

Supplement: Additional file 11 — Results of SAMOVA analyses (Pyrenees matrix). [file 1471-2148-14-146-S11.pdf]

ITS

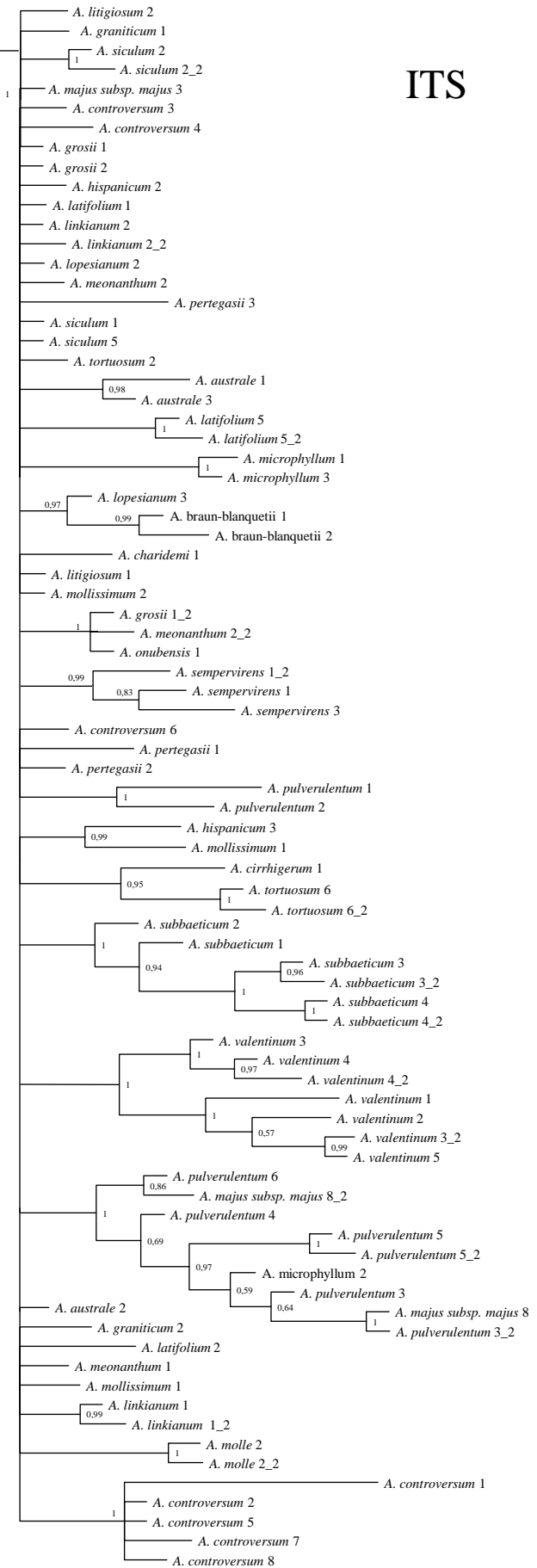

Supplement: Additional file 12 — Bayesian phylogenetic tree constructed with nrITS sequences published in 22plus the five new nrITS sequences corresponding to the samples collected across South-western Alps for this study. [file 1471-2148-14-146-S12.pdf]
