# Supplementary material for: The evolutionary history of Antirrhinum in the Pyrenees inferred from phylogeographic analyses
Source: BMC Evol Biol. 2014 Jun 26;14:146. doi: 10.1186/1471-2148-14-146 (PMC4099501; doi:10.1186/1471-2148-14-146)
Supplement: Additional file 14 — Methods. [file 1471-2148-14-146-S14.doc]

**Additional file 14**

*Phylogenetic analysis (Antirrhinum matrix)*

Phylogenetic analyses for 190 *trn*S-*trn*G/*trn*K-*mat*K concatenated sequences were conducted using Bayesian inference (BI), maximum likelihood (ML) and maximum parsimony (MP). In addition, Bayesian phylogenetic analyses were also performed on the separate matrices to examine plastid gene tree congruence. *Gambelia speciosa* and *Misopates orontium* were selected as the outgroup based on previous phylogenetic evidence , and gaps were treated as missing data. The MP analysis was performed in TNT 1.1 using a heuristic search with 10,000 replicates saving two most-parsimonious trees per replicate, followed by a second heuristic search retaining all best trees and using the trees obtained in the previous 10,000 replicates as the starting ones. Bootstrap support (MP-BS) of clades was assessed using 1000 standard replicates. For ML and BI analyses, the simplest model of sequence evolution that best fits the sequence data was determined under the Akaike Information Criterion (AIC) in jModeltest 0.1.1 . The General Time Reversible model incorporating invariant sites and a gamma distribution (GTR+I+G) was selected for the two plastid DNA regions. ML was implemented in PhyML 3.0 with 500 non-parametric bootstrap replicates (ML-BS). BI was performed in MrBayes v3.1.2 . Two identical searches with 10 million generations each and a sample frequency of 1000 were performed. Chain convergence was assessed with Tracer 1.5 , and a 50% majority rule consensus tree with Bayesian posterior probabilities (PP) of clades was calculated, using the sumt command, to yield the final Bayesian estimate of phylogeny after removing the first 10% generations as burn-in. Trees were visualized using FigTree 1.3.1. .

*Ancestral area reconstructions (Antirrhinum matrix)*

A discrete phylogeographic analysis (DPA) that uses standard MCMC sampling implemented in BEAST was performed to assess the probability distribution of the geographic locations in each node of the maximum clade credibility tree. A total of 14 discrete areas were delimited: (i) the four Iberian quadrants (northeastern Iberia, NE; northwestern Iberia, NW; southeastern Iberia, SE; southwestern Iberia, SW), as divided by the geographical coordinates 40ºN/5ºW ; (ii) Eastern, Central and Western Pyrenees, as the three recognized biogeographic regions within the Pyrenees (see below); (iii) the other two northern areas sampled nearby the Pyrenees (Southern French basin and South-western Alps); and (iv) the remaining five regions sampled across Mediterranean basin (Morocco, Sicily, Sardinia, Italy and Turkey). Statistical significance for the rates of the dispersal events was assessed via Bayes factor test (BF) as described by Lemey *et al.* . Dispersal rates were allowed to be zero with some probability in the framework of Bayesian stochastic search variable selection (BSSVS). The analysis consisted of two independent runs of 100 million generations each sampling every 10000 generations. Chain convergence was examined in Tracer 1.5 . The two runs were combined in LogCombiner 1.6.2 after discarding the first 10% of sampled generations as burn-in. A consensus chronogram with the maximum sum of clade credibilities (MCC), was obtained with TreeAnotator v.1.6.2 and visualized in FigTree 1.3.1 . Well-supported rates of dispersal (BF>3) were visualized in Google Earth using the RateIndicatorBF tool added to the BEAST code.

Additional ancestral range reconstructions were conducted using the Bayesian time-calibrated molecular phylogeny with the aim to discriminate between northern and southern origin of Pyrenean lineages.  For this purpose, only four areas were delimited (i) Iberian Peninsula, (ii) Pyrenees and adjacent areas, (iii) South-western Alps, and (iv) samples from the Mediterranean basin (excluding Iberia). Ancestors were allowed to be present in all of them. Distribution ranges of sequences (haplotypes) instead of species was used . Two alternative reconstruction methods were used: (a) [statistical dispersal-vicariance analysis](http://www.sciencedirect.com/science/article/pii/S105579031000165X) (S-DIVA) implemented in the program RASP 1.1 , a parsimony-based approach (DIVA; ) that determines the probability of each geographical region for each node, accounting for the uncertainty of the Bayesian phylogenetic analysis ; and (b) dispersal-extinction-cladogenesis analysis (DEC) implemented in the software package Lagrange v2.0.1 , a parametric likelihood-based approach that estimates the most likely geographic distribution of two daughter lineages following a speciation event. Whereas the first method estimates the actual state at the node, the second estimates the states of the branches emanating from a given node. For the S-DIVA analysis we followed the method of Harris & Xiang . Two hundred trees randomly sampled after the burn-in period from the BEAST run were selected, and the single MCC tree was used as final tree (after pruning outgroup taxa). For DEC analysis we used the pruned MCC tree. Symmetric dispersal between both areas and constant dispersal rates through time were set.

*Genetic diversity and geographic structure (Pyrenees matrix)*

An analysis of genetic diversity was carried out across the three recognized biogeographic regions in which the Pyrenees range is divided (Eastern, Central and Western Pyrenees) (see Fig. 3a). The boundaries of this three biogeographic areas, although with slight differences, have been traditionally established by both geologists and phytogeographers on the basis of geologic, climatic and floristic data. Haplotype frequencies and molecular diversity indices for each biogeographic area were calculated using DnaSP v5 . In addition, to identify potential hotspots of genetic diversity across the Pyrenees, individuals were geographically grouped by means of a 10x10 km grid. Charts representing haplotype frequencies were constructed for each grid cell, which was named by a generic letter–number code (Fig. 3).

To infer the spatial genetic structure we used a Bayesian model-based approach, implemented in the BAPs software, version 5.3 . This software assigns the genotypes into genetically structured groups (K) and incorporates the possibility to account for the dependence due to linkage between the sites within aligned sequences. Five iterations of K, for Kmax values of five, ten and 20 potential populations, were conducted to determine the optimal number of genetically homogeneous groups. ‘Clustering of groups with linked loci’ analysis was chosen, and the groups were defined by natural sampled populations. Admixture analyses were run with 100 iterations to estimate admixture coefficients for individuals, 200 reference individuals from each population and 20 iterations to estimate admixture coefficients for reference individuals.

To identify genetic subdivisions among the Eastern, Central and Western Pyrenees, we performed an analysis of molecular variance (AMOVA) , which compares haplotype variation within and between groups. Pairwise F_ST_ statistics were also calculated to estimate genetic distances. Both analysis were performed by using ARLEQUIN . Additionally, an AMOVA was performed in order to assess the partitioning of variance between the Lineage E (primarily distributed in the eastern part of the Pyrenees) and the rest of lineages (see below).

To evaluate the optimal grouping of the sampled sites without a priori assumptions, a spatial analysis of molecular variance (SAMOVA) implemented in the software package SAMOVA 1.0 was also performed. This analysis uses a simulated annealing approach based on genetic and geographical data to identify groups of related populations. The program was run for K = 2 to 20 groups, from 100 initial conditions, and the most likely structure was identified using highest values of F_CT_ (the proportion of genetic variation between groups of populations) excluding any groups of a single population.

**References**
